# Non-eosinophilic esophagitis eosinophilic gastrointestinal diseases are more prevalent than eosinophilic esophagitis in Korean children: from a multicenter study based on new diagnostic criteria and nomenclature

**DOI:** 10.3389/fped.2025.1656107

**Published:** 2025-08-14

**Authors:** Kunsong Lee, Ben Kang, Eun Sil Kim, Dae Yong Yi, Tae Hyeong Kim, Yoo Min Lee, Sujin Choi, Byung-Ho Choe

**Affiliations:** ^1^ Department of Pediatrics, Dankook University College of Medicine, Cheonan, Republic of Korea; ^2^ Department of Pediatrics, School of Medicine, Kyungpook National University, Daegu, Republic of Korea; ^3^ Department of Pediatrics, Kangbuk Samsung Hospital, Sungkyunkwan University School of Medicine, Seoul, Republic of Korea; ^4^ Department of Pediatrics, Chung-Ang University Hospital, Chung-Ang University, College of Medicine, Seoul, Republic of Korea; ^5^ Department of Pediatrics, Kyung Hee University Hospital at Gangdong, Seoul, Republic of Korea; ^6^ Department of Pedaitrics, Soonchunhyang University Bucheon Hospital, Soonchunhyang University College of Medicine, Bucheon, Republic of Korea

**Keywords:** eosinophilic gastrointestinal diseases (EGIDs), eosinophilic esophagitis (EoE), eosinophilic gastritis (EoG), pediatrics, multicenter study, nomenclature, guideline

## Abstract

**Background:**

Eosinophilic gastrointestinal diseases (EGIDs) are chronic inflammatory disorders characterized by eosinophilic infiltration of the gastrointestinal (GI) tract. This study aimed to investigate the epidemiological and clinical characteristics of pediatric EGIDs in Korea based on the newly established nomenclature and diagnostic guidelines.

**Methods:**

A retrospective analysis was conducted on pediatric patients (0–18 years) with GI symptoms who underwent upper and lower GI endoscopy at five tertiary hospitals in Korea (2010∼2023). Patients were classified based on the latest diagnostic criteria into eosinophilic esophagitis (EoE) and non-eosinophilic esophagitis eosinophilic gastrointestinal disease (non-EoE EGIDs), including eosinophilic gastritis (EoG), eosinophilic enteritis (EoN), and eosinophilic colitis (EoC). Clinical, laboratory, and endoscopic findings were analyzed.

**Results:**

Among a total of 4,972 pediatric endoscopic procedures (3,300 upper and 1,672 lower), 63 cases (1.3%) of EGIDs were diagnosed, with non-EoE EGIDs (65.1%) being more prevalent than EoE (34.9%). Within the non-EoE EGIDs group, EoG was the most common subtype (33.3%), followed by EoN (20.6%) and EoC (7.9%). Multi-site involvement was observed in 30.2% with frequent esophageal involvement (EI). Endoscopic abnormalities, including rings and furrows in EoE and ulcers in non-EoE EGIDs, were common. Peripheral eosinophil counts and fecal calprotectin levels were significantly higher in non-EoE EGIDs with EI than in EoE (*P* < 0.05). The relapse rates exceeded 20% across all subtypes.

**Conclusion:**

This study highlights the distinctive epidemiology of pediatric EGIDs in Korea, where non-EoE EGIDs are more prevalent than EoE, contrasting with Western reports. Peripheral eosinophil counts and fecal calprotectin levels were significantly higher in non-EoE EGIDs with EI than in EoE.

## 1 Introduction

Eosinophilic gastrointestinal diseases (EGIDs) are characterized by eosinophilic infiltration and dysfunction of the gastrointestinal (GI) tract and they are classified into eosinophilic esophagitis (EoE) and non-eosinophilic esophagitis eosinophilic gastrointestinal diseases (non-EoE EGIDs) based on the affected site, with EoE being the most extensively studied subtype (1). Since the establishment of diagnostic guidelines for EoE in 2007 (2), numerous studies have been conducted on its clinical features, endoscopic findings, treatment options, and prognosis. The incidence of EoE has been reported at 5.31 cases per 100,000 individuals, with a prevalence of 40.4 cases per 100,000 — a pattern more commonly observed in North America and Europe than in Asia (3).

In contrast, non-EoE EGIDs have historically lacked standardized diagnostic criteria, resulting in limited recognition and research on these conditions. To address this gap, a new nomenclature system for EGIDs was introduced in 2022 (1). According to this updated nomenclature, EGIDs are classified into EoE and non-EoE EGIDs based on the affected site: eosinophilic gastritis (EoG), eosinophilic enteritis (EoN), and eosinophilic colitis (EoC). In cases involving multiple GI sites, the primary site is designated first, followed by secondary sites (e.g., eosinophilic gastritis and enteritis). However, concurrent esophageal involvement (EI) remains a subject of debate; some experts propose terms such as “EoG with esophageal involvement” or “EoG with EoE” (1). Unlike the esophagus, other GI organs naturally contain eosinophils as part of their innate immune system**.** This intrinsic presence of eosinophils in these organs complicates efforts to define pathological thresholds for abnormal eosinophilic infiltration in non-EoE EGIDs. Additionally, nonspecific symptoms such as abdominal pain or diarrhea further challenge accurate diagnosis.

In 2024, guidelines for diagnosing and managing pediatric non-EoE EGIDs were published (4). These guidelines aim to improve recognition of these conditions by providing clear thresholds for histological diagnosis and recommendations for clinical management. Despite these advancements, updated epidemiological studies on pediatric EGIDs remain limited. Before the introduction of the new guidelines, a prevalence study on non-EoE EGIDs reported that approximately 1.9% of patients presenting with gastrointestinal symptoms were diagnosed with non-EoE EGIDs (5). While a nationwide multi-center study on EoE among Korean children has been conducted (6), comprehensive research encompassing both EoE and non-EoE EGIDs has yet to be performed.

To address this gap in knowledge, this study aims to investigate the epidemiological patterns and clinical characteristics of pediatric EGIDs in Korea based on the newly established nomenclature and guidelines.

## 2 Materials and methods

### 2.1 Study population

From January 2010 to July 31, 2023, pediatric patients (aged 0–18 years) who underwent upper gastrointestinal (GI) endoscopic procedures (*n* = 3,300) or lower GI endoscopic procedures (*n* = 1,672) at five tertiary hospitals in Korea due to gastrointestinal symptoms were retrospectively reviewed. Patients diagnosed with EGIDs based on the established diagnostic guidelines^4^ were included in the study.

### 2.2 Diagnostic criteria

According to the nomenclature proposed by Dellon et al. (7), EGIDs were classified into EoE, EoG, EoN, and EoC. For small intestinal evaluation, duodenal biopsies were routinely obtained during upper endoscopy and terminal ileal biopsies were obtained during lower endoscopy when indicated. However, jejunal biopsies were not performed due to procedural restrictions in children. In cases with duodenal or ileal eosinophilic infiltration were designated as eosinophilic duodenitis (EoD) or eosinophilic ileitis (EoI), respectively, and were overall classified as eosinophilic enteritis (EoN) in accordance with the latest nomenclature.

In cases where eosinophilic infiltration involved multiple gastrointestinal sites, the primary affected site was designated first, followed by the secondary site, such as EoG and Eosinophilic duodenitis (EoD) or EoD and EoC. If secondary esophageal involvement was present, it was indicated as “with esophageal involvement (EI)”.

For pathological diagnosis, EoE was diagnosed based on the existing guidelines (2). Non-EoE EGIDs were diagnosed following the criteria established by Papadopoulou et al. (4), requiring eosinophil counts exceeding the established threshold in high-power field microscopy and the absence of evidence for other conditions causing eosinophilic infiltration.

### 2.3 Data collection and analysis

This study was conducted as a retrospective observational study, collecting data from multiple institutions to investigate the overall prevalence of EGIDs, as well as the clinical characteristics, endoscopic findings, and treatment patterns according to EGIDs subtypes.

### 2.4 Analysis

Continuous variables were expressed as medians with interquartile ranges (IQRs) and analyzed using the Mann–Whitney U test. Comparisons of continuous variables across multiple EGIDs subtypes were performed using the Kruskal–Wallis test. Categorical variables were analyzed using Fisher's exact test. Statistical significance was set at *P* < 0.05.

All statistical analyses were performed using SPSS 28 (IBM Corp., *IBM* SPSS Statistics for Windows, Version 28.0, Armonk, NY: IBM Corp; 2021).

### 2.5 Ethical statement

This study was approved by the Institutional Review Board of Dankook University Hospital (IRB No. 2023-07-007).

## 3 Results

### 3.1 Prevalence and epidemiological characteristics of EGIDs

Among the 3,300 upper gastrointestinal endoscopic procedures and 1,672 lower gastrointestinal endoscopic procedures performed in children, 58 cases (1.8%) of EGIDs—excluding EoC—were diagnosed from upper endoscopic examinations, and 5 cases (0.3%) of EoC were diagnosed from lower endoscopic examinations.

The total number of EGID diagnoses was 63 (based on procedure counts), with a marked male predominance (male-to-female ratio: 2.9:1; 47 males, 16 females), and a mean age at diagnosis of 8.29 ± 5.19 years.

**Figure 1:**
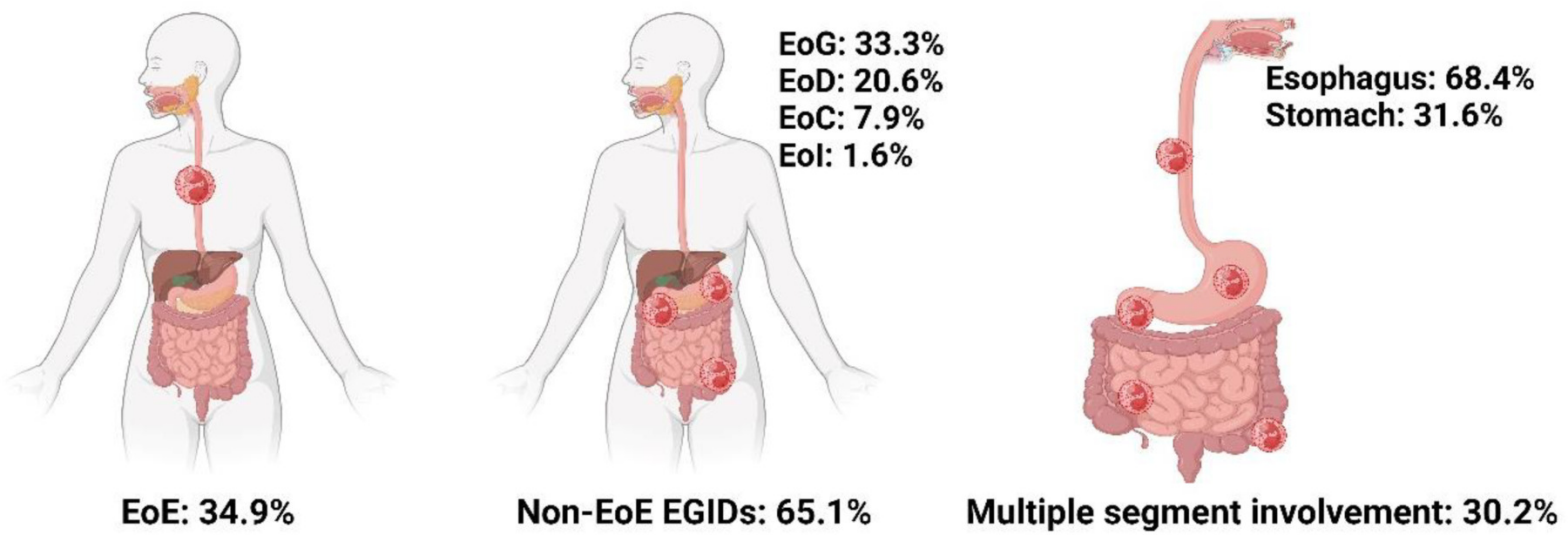
Epidemiologic characteristics of distribution and involvement sites in EGIDs. Created in BioRender. Lee, K. (2025) https://BioRender.com/y56g240.

**Figure 2:**
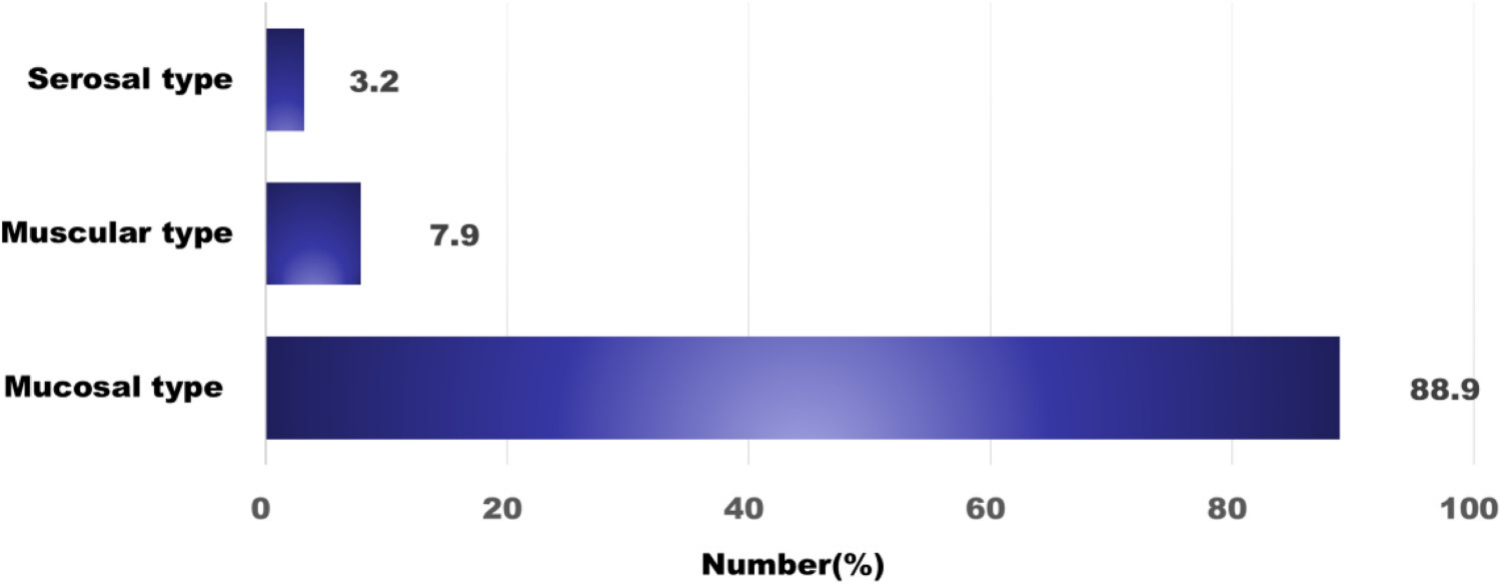
The classification based on the extent of eosinophil infiltration. Mucosal invasion is the most common.

Regarding subtype distribution, EoE accounted for 34.9% of diagnosed cases, while non-EoE EGIDs made up 65.1%. Within the non-EoE group, EoG represented 33.3%, EoD 20.6%, EoC 7.9%, and eosinophilic ileitis (EoI) 1.6% (Figure 1). Multiorgan involvement was observed in 30.2% of diagnoses. When secondary sites were considered, the esophagus was most frequently affected (68.4%), followed by the stomach (31.6%). Histologically, the mucosal form was predominant (88.9%), with muscular (7.9%) and serosal (3.2%) types being less common (Figure 2).

### 3.2 Clinical analysis by EGIDs subtypes

At the time of diagnosis, the median ages were as follows: EoE, 10 years; EoG, 9 years; EoN, 13 years; and EoC, 9 years. Laboratory assessments revealed that median total IgE concentrations and peripheral eosinophil counts were highest in EoC cases, although these differences did not reach statistical significance (Table 1).

**Table 1:** Comparative analysis of clinical features by eosinophilic gastrointestinal diseases classification.

Variable	EoE (*n* = 22)	EoG (*n* = 21)	EoN (*n* = 15)	EoC (*n* = 5)	*P-* value
Age at diagnosis, median [IQR]	10 [1–16]	9 [1–15]	13 [1–17]	9 [9–15]	0.705
Male, *n* (%)	18 (81.8)	14 (66.7)	12 (80.0)	3 (60.0)	0.610
Female, *n* (%)	4 (18.2)	7 (33.3)	3 (20.0)	2 (40.0)	
Total IgE (IU/ml), median [IQR]	43 [42–1,561]	232 [66–5,000]	234 [8–1,640]	426 [31–1,650]	0.764
Blood eosinophil count (/μl), median [IQR]	315 [93–5,794]	650 [189–1,478]	199 [65–21,148]	1,111[529–8,973]	0.376
Calprotectin (mg/kg), median [IQR]	69 [4–740]	296 [12–3,676]	74 [5–346]	10 [0.3–197]	0.330
Allergic diseases, *n* (%)	12 (54.5)	9 (42.8)	8 (53.3)	5 (100.0)	0.571
Atopic dermatitis, *n* (%)	3 (13.6)	5 (23.8)	3 (20.0)	2 (40.0)	0.516
Food allergy, *n* (%)	7 (31.8)	3 (14.2)	1 (6.6)	3 (60.0)	0.147
Asthma, *n* (%)	6 (27.2)	3 (14.2)	2 (13.3)	2 (40.0)	0.753
Rhinitis, *n* (%)	7 (31.8)	5 (23.8)	6 (40.0)	3 (60.0)	0.803
Symptoms, *n* (%)					
Abdominal pain, *n* (%)	12 (54.5)	13 (61.9)	11 (73.3)	3 (60.0)	0.756
Weight loss, *n* (%)	2 (9.1)	5 (23.8)	3 (20.0)	3 (60.0)	0.678
Diarrhea, *n* (%)	0 (0.0)	1 (4.8)	3 (20.0)	2 (40.0)	**0**.**012**
Vomiting, *n* (%)	12 (54.5)	10 (47.6)	10 (66.7)	4 (80.0)	0.498
Anemia, *n* (%)	0 (0.0)	4 (19.0)	0 (0.0)	0 (0.0)	0.058
Relapse, *n* (%)^†^	7 (35.0)	5 (23.8)	3 (20.0)	2 (40.0)	0.712
Eosinophil involvement site, *n* (%)					**<0**.**001**
Esophagus, *n* (%)	22 (100.0)	7 (33.3)	5 (33.3)	1 (20.0)	
Stomach, *n* (%)	0 (0.0)	21 (100.0)	5 (33.3)	1 (20.0)	
Small intestine, *n* (%)	0 (0.0)	0 (0.0)	15 (100.0)	0 (0.0)	
Colon, *n* (%)	0 (0.0)	0 (0.0)	0 (0.0)	5 (100.0)	

The bold values indicate statistical significance.

EoE, eosinophilic esophagitis; EoG, eosinophilic gastritis; EoN, eosinophilic enteritis; EoC, eosinophilic colitis.

^†^ Relapsing cases were identified among patients who had been followed up for more than 12 months.

Among presenting symptoms, diarrhea was notably absent in EoE but was reported in 40% of EoC cases, a difference that was statistically significant (*P* = 0.012) (Table 1). For cases with at least one year of follow-up, the rate of symptom recurrence exceeded 20% across all subtypes.

Evaluation of organ involvement showed that 33% of EoG cases also had concurrent EI. In the EoN group, one-third of cases exhibited both esophageal and duodenal involvement, and another third had simultaneous stomach and duodenum involvement. In EoC, simultaneous eosinophilic infiltration of the esophagus or stomach was observed in one case each (Table 1).

### 3.3 Comparative analysis between EoE and non-EoE EGIDs with esophageal involvement

When comparing EoE to non-EoE EGIDs with EI, the latter group demonstrated significantly higher median peripheral eosinophil counts at diagnosis (363/μl vs. 316/μl, *P* = 0.031) and elevated median fecal calprotectin levels (109.5 mg/kg vs. 69.0 mg/kg, *P* = 0.045). Additionally, weight loss was reported more frequently among non-EoE EGIDs affecting the esophagus (*P* = 0.019) (Table 2).

**Table 2:** Comparative analysis between EoE and non-EoE EGIDs with esophageal involvement.

Variable	EoE (*n* = 22)	Non-EoE EGIDs with EI (*n* = 13)	*P*-value
Age at diagnosis, median [IQR]	10 [1–16]	9 [1–11]	0.117
Male, *n* (%)	18 (81.8)	12 (92.3)	0.630
Female, *n* (%)	4 (18.2)	1 (7.7)	
Total IgE (IU/ml), median [IQR]	429 [42–1,561]	780 [208–3,000]	0.441
Blood eosinophil count (/μl), median [IQR]	316 [93–5,794]	363 [180–5,787]	0.031
Calprotectin (mg/kg), median [IQR]	69.0 [3.8–739.9]	109.5 [3.0–3,676]	0.045
EREFS, median [IQR]	2.0 [ 0.0–6.0]	2.0 [0.0–6.0]	0.310
Allergic diseases, *n* (%)	12 (54.5)	5 (38.4)	0.683
Atopic dermatitis, *n* (%)	3 (13.6)	1 (7.7)	1.000
Food allergy, *n* (%)	7 (31.8)	2 (15.4)	0.667
Asthma, *n* (%)	6 (50.0)	2 (15.4)	0.676
Rhinitis, *n* (%)	7 (31.8)	4 (33.3)	1.000
Symptoms, *n* (%)			
Abdominal pain, *n* (%)	12 (54.5)	9 (69.2)	0.448
Weight loss, *n* (%)	2 (9.1)	6 (46.2)	0.019
Diarrhea, *n* (%)	0 (0.0)	1 (7.7)	0.371
Vomiting, *n* (%)	12 (54.5)	7 (53.8)	1.000
Relapse, *n* (%)	7 (31.8)	4 (30.7)	1.000

IQR, interquartile range; EoE, eosinophilic esophagitis; Non-EoE EGIDs with EI, non-eosinophilic esophagitis eosinophilic gastrointestinal diseases with esophageal involvement; EREFS, endoscopic reference score.

### 3.4 Endoscopic findings analysis

Endoscopic evaluation revealed that normal mucosal appearance was most commonly seen in EoN (40%), followed by EoG (23.8%), EoE (22.7%), and EoC (20%). In EoE, rings and furrows were the most common endoscopic findings, observed in 59.1% of cases. Alternatively, ulcers or erosions (60%) were identified as the predominant endoscopic features in non-EoE EGIDs. Erythema was the most frequent endoscopic finding in EoG (42.9%), and pyloric stenosis was observed in 19% of the EoG cases. The mean EoE Endoscopic Reference Score (EREFS) at diagnosis was higher in the non-EoE EGIDs with EI group (2.69 ± 1.75) compared to the EoE group (2.05 ± 1.73), although this difference did not reach statistical significance (Table 3).

**Table 3:** Endoscopic findings of EoE and non-EoE EGIDs at diagnosis.

Endoscopic finding	EoE (*n* = 22)	EoG (*n* = 21)	EoN (*n* = 15)	EoC (*n* = 5)
Edema	7 (31.8%)	–	–	–
Rings	13 (59.1%)	–	–	–
Exudates	5 (22.7%)	–	–	–
Furrows	13 (59.1%)	–	–	–
Stricture	0 (0.0%)	–	–	–
Normal	5 (22.7%)	5 (23.8%)	6 (40.0%)	1 (20.0%)
Ulcer or erosions	–	5 (23.8%)	9 (60.0%)	3 (60.0%)
Granularity	–	4 (19.0%)	0 (0%)	0 (0.0%)
Nodularity	–	2 (9.5%)	0 (0%)	0 (0.0%)
Erythema	–	9 (42.9%)	0 (0%)	1 (20.0%)
Friability	–	2 (9.5%)	0 (0%)	0 (0.0%)
Fold thickening	–	3 (14.3%)	0 (0%)	0 (0.0%)
Pyloric stenosis	–	4 (19.0%)	–	–

EoE, eosinophilic esophagitis; EoG, eosinophilic gastritis; EoN, eosinophilic enteritis; EoC, eosinophilic colitis. Data are presented as number (%). “–” indicates the absence of the finding or non-applicability.

### 3.5 Analysis of treatment trends

**Figure 3:**
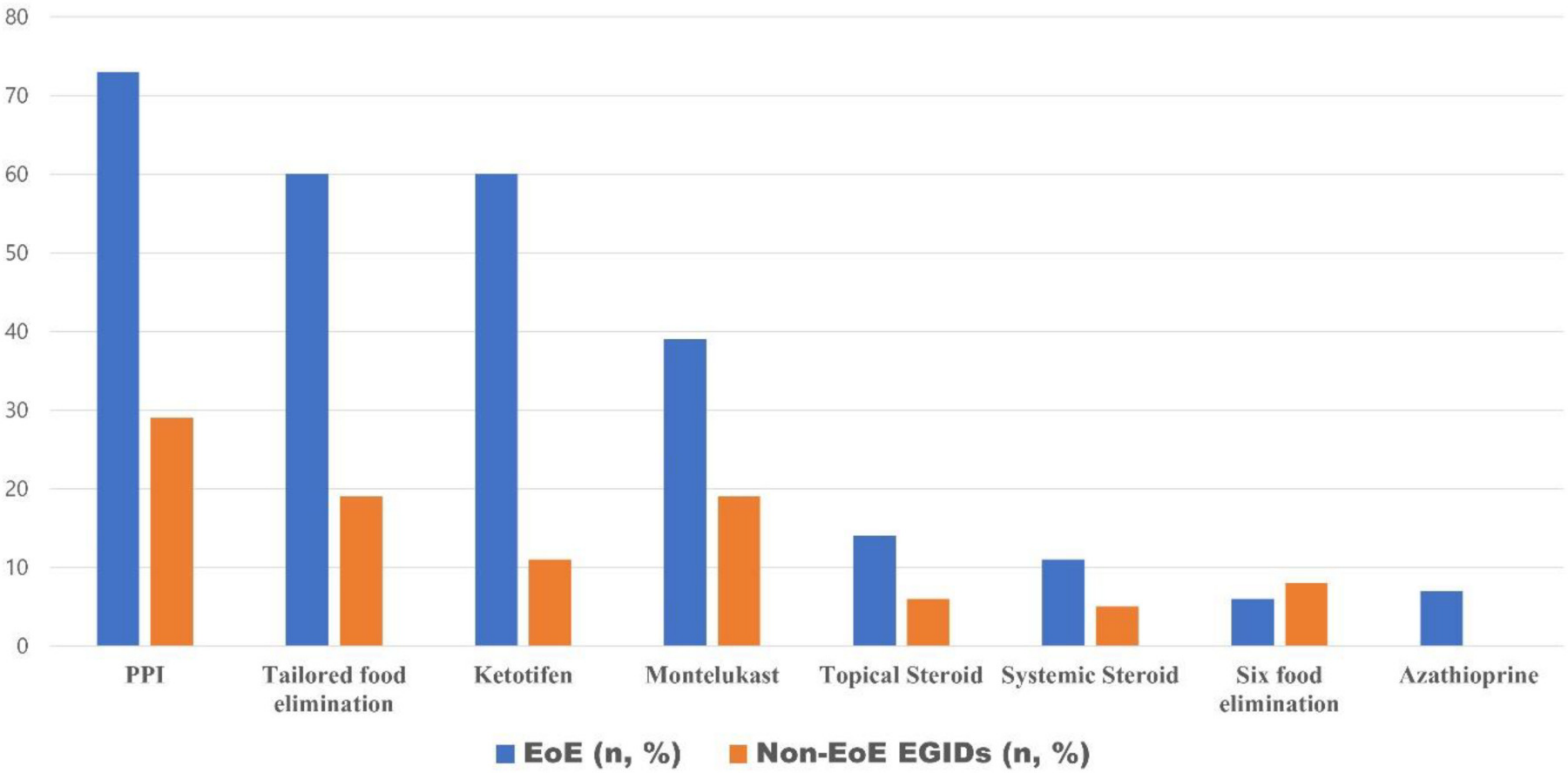
The current status of treatment for pediatric eosinophilic gastrointestinal diseases in Korea.

Analysis of therapeutic approaches indicated that proton pump inhibitors (PPIs) were the most frequently prescribed medication, used in 73% of EoE cases and 29% of non-EoE EGIDs cases. Individualized elimination diets were implemented in 59% of EoE cases and 20% of those with non-EoE EGIDs. Other pharmacologic interventions, in order of decreasing frequency, included ketotifen, montelukast, topical corticosteroids, systemic corticosteroids, and azathioprine. Six-food elimination diets were employed in 5% of EoE and 7% of non-EoE EGIDs cases (Figure 3).

## 4 Discussion

Multiple studies have established that EoE is no longer considered a rare disease. However, non-EoE EGIDs are still regarded as significantly rarer compared to EoE. This may be attributed to the fact that, unlike the esophagus, normal eosinophilic infiltration can be observed in other GI organs. Furthermore, even when abnormal eosinophilic infiltration is detected, the diagnosis of non-EoE EGIDs requires the exclusion of various other conditions that can lead to eosinophilic infiltration of the GI tract, such as inflammatory bowel disease and functional gastrointestinal disorders. Consequently, determining the pathological threshold for eosinophilic infiltration and making a definitive diagnosis of non-EoE EGIDs remain inherently challenging.

Despite these challenges, the incidence and prevalence of non-EoE EGIDs are increasing (8). According to a study by Jensen et al. (9), environmental factors such as a history of neonatal intensive care unit (NICU) admission, antibiotic exposure during infancy, and maternal complications during pregnancy have been associated with the epidemiological shift in EGIDs, similar to findings in EoE.

Before the establishment of formal diagnostic guidelines and nomenclature for pediatric non-EoE EGIDs, a meta-analysis assessing the pooled overall prevalence of non-EoE EGIDs reported that 1.9% of adults and pediatric patients presenting with GI symptoms were diagnosed with non-EoE EGIDs (9). In this study, a total of 4,972 endoscopic procedures (3,300 upper and 1,672 lower) were performed in children. EGIDs were diagnosed in 58 cases (1.8%) based on upper endoscopic findings and 5 cases (0.3%) based on lower endoscopic findings. The prevalence of EoE was 0.7% among upper endoscopic procedures, while non-EoE EGIDs (excluding EoC) accounted for 1.1%. It should be noted that since some patients underwent both upper and lower GI endoscopy, the procedure count exceeds the number of unique individuals, and thus, prevalence rates may be slightly overestimated compared to patient-based calculations.

Epidemiological studies from the United States and Europe generally report a higher prevalence of EoE compared to non-EoE EGIDs. However, systematic reviews of studies published in Asia have consistently shown that the prevalence of non-EoE EGIDs is higher than that of EoE in Eastern populations (10). Interestingly, a single-center study from Italy on pediatric EGIDs reported that non-EoE EGIDs were more prevalent than EoE (5.1% vs. 4.4%) (11). This suggests that the epidemiological and clinical characteristics of EGIDs may vary based on demographic and environmental factors specific to each region (10, 12).

However, the authors fundamentally believe that the primary reason for the observed epidemiological differences is that EoE has well-established diagnostic criteria, whereas non-EoE EGIDs have historically lacked standardized diagnostic guidelines. As a result, the diagnosis of non-EoE EGIDs may have been relatively underrecognized, leading to an underestimation of its true prevalence. With the newly established diagnostic criteria for non-EoE EGIDs, future large-scale epidemiological studies are expected to provide objective evidence regarding the epidemiological characteristics of EGIDs as a whole. Although this study is not a large-scale investigation, it holds significance as a foundational study contributing to the establishment of evidence in this field.

When comparing the findings of this study with previous research, a nationwide hospital-based survey conducted by Yamamoto et al. (12) in Japan, which included both adult and pediatric patients with EGIDs, reported a diagnostic distribution of 39% for EoE and 61% for non-EoE EGIDs. These results are comparable to the findings of the present study, in which EoE accounted for 34.9% and non-EoE EGIDs for 65.1%. In contrast, a cross-sectional study conducted in the United States (13) demonstrated a significantly higher prevalence of EoE (73.4%) compared to non-EoE EGIDs with or without concomitant EoE (26.6%), confirming a clear predominance of EoE in Western populations. Additionally, this study reported that EoE was more common in males, whereas non-EoE EGIDs were more frequently observed in females. However, in the present study, both EoE and non-EoE EGIDs were more prevalent in males.

A recent population-based study conducted in Japan investigated the incidence and prevalence of non-EoE EGIDs. As of 2022, the incidence and prevalence were reported to be 3.07 per 100,000 individuals per year and 17.23 per 100,000 individuals, respectively (8). Consistent with findings from other studies, both the incidence and prevalence of non-EoE EGIDs have shown a steady increase over time. Furthermore, compared to the reported prevalence of non-EoE EGIDs in Western populations (ranging from 2 to 8 per 100,000 individuals), the prevalence in Japan was notably higher. This reinforces the observation that non-EoE EGIDs are more dominant in Asian populations (3, 5, 13–15). According to the findings of Shoda et al. (16), variations in genetic background, environmental exposures, and the composition of the gut microbiome may help explain the distinct epidemiological patterns of EGIDs observed in Eastern and Western populations. Nevertheless, as large-scale comparative studies investigating the underlying causes of these regional differences are still lacking, further research will be essential to fully elucidate these mechanisms.

The symptoms of EGIDs vary depending on the location and extent of eosinophilic infiltration within the GI tract. Since most symptoms are confined to the mucosa of the affected organ, they are often nonspecific (17). In mucosal type non-EoE EGIDs, epithelial disruption caused by ulcers or erosions leads to symptoms such as abdominal pain, dyspepsia, or bleeding due to tissue damage. In muscular type involvement, eosinophilic infiltration into the muscularis layer can result in symptoms of obstruction, including vomiting and abdominal distension (17). Serosal type EGIDs involve deeper penetration into the intestinal wall, often presenting with peripheral eosinophilia, massive eosinophilic ascites, and associated symptoms such as abdominal distension and pain (17).

In the present study, mucosal type EGIDs accounted for 88.9% of all cases. Muscular type involvement, characterized by antral obstruction, was observed in 7.9% of cases. Serosal type EGIDs had a prevalence of 3.2% and were associated with massive peripheral eosinophilia (with a peak eosinophil count of 21,148/μl) and eosinophilic ascites. All serosal type cases met the diagnostic criteria for EoC based on endoscopic biopsy findings. Notably, these cases showed an immediate response to systemic steroid therapy and exhibited no recurrence after more than 20 months of follow-up.

According to a study by Persek et al. (18), multifocal eosinophilic infiltration beyond the primary site of involvement was observed in 41% of patients with non-EoE EGIDs. The esophagus was reported as the most commonly affected secondary site, and pediatric patients were found to have a higher tendency for multifocal GI involvement compared to adults. Similarly, in the present study, 30.2% of cases showed eosinophilic infiltration in two or more GI organs. The esophagus (68.4%) was the most commonly involved secondary site, followed by the stomach (31.6%).

In our comparative analysis between the EoE group and the non-EoE EGIDs group with EI, both groups demonstrated a male predominance (80%). However, peripheral eosinophil counts and calprotectin levels were significantly higher in the non-EoE EGIDs with EI group compared to the EoE group. Similarly, a pediatric cohort study on EGIDs conducted in the United States (17) found that both peripheral blood eosinophil counts and eosinophil counts in esophageal tissue were higher in the non-EoE EGIDs with EI group than in the EoE group. Additional studies have also reported higher peripheral eosinophil counts in patients with non-EoE EGIDs compared to those with EoE (10, 12, 17). In this study, although the number of cases with serosal type EGIDs accompanied by ascites was limited, making it difficult to draw definitive conclusions, peripheral eosinophil counts were noticeably higher in serosal type cases compared to mucosal type and muscular type EGIDs.

According to the non-EoE EGIDs guidelines (4), the use of calprotectin for diagnosis and disease activity assessment is not recommended. However, in this study, calprotectin levels were significantly higher in the non-EoE EGIDs with EI group compared to the EoE group. Yoo et al. (19), also reported that fecal calprotectin is a useful non-invasive biomarker in Korean children, supporting its clinical utility for differentiating organic gastrointestinal diseases from functional abdominal pain disorders. This finding suggests that eosinophil-driven inflammation may be more widely distributed across multiple GI sites beyond the esophagus in non-EoE EGIDs with EI. To minimize the potential influence of alternative intestinal disorders on peripheral eosinophil counts and fecal calprotectin levels, all patients with suspected EGIDs underwent both upper and, when clinically indicated, lower gastrointestinal endoscopy. Systematic biopsies were obtained from the terminal ileum and colon as needed. Nevertheless, the retrospective nature of the study may not entirely eliminate the possibility of unrecognized or subclinical alternative gastrointestinal inflammation.

Additionally, a study by Rohani et al. (20) demonstrated a decrease in calprotectin levels following elimination diet therapy in patients with EoC, suggesting its potential utility as a biomarker for treatment response. However, to establish calprotectin as a reliable marker for non-EoE EGIDs, further large-scale studies are warranted (19, 21).

Studies on relapse and prognosis in non-EoE EGIDs remain limited. However, a study by Quinn et al. (22) reported that 25% of patients with EoG or EoD experienced persistent or recurrent eosinophilic infiltration. Additionally, they found an association between endoscopic scores at the time of EoG diagnosis and the likelihood of disease recurrence. In the present study, among patients with at least 12 months of follow-up, the relapse rate was 35% in EoE, 23.8% in EoG, 20.0% in EoN, and 40% in EoC, However, due to the limited number of cases, statistical significance could not be established.

Studies have reported that despite active EGIDs, up to 25% of EoE cases and up to 50% of EoG cases may present with normal endoscopic findings (23). When comparing the results of previous studies with the findings of this study, 22.7% of EoE cases in this study showed normal endoscopic findings. The most common abnormalities were furrows and rings (59.1%), while strictures were not observed. In Western populations, esophageal stenosis has been reported in 17.5% of adults and 2.5% of pediatric patients (24). However, similar to the present study, previous studies in Asian populations have reported a lower prevalence of fixed concentric rings and stenosis compared to Western populations (25). In this study, there was no significant difference in endoscopic scores between the EoE group and the non-EoE EGIDs with EI group, a finding that has also been reported in other studies (25).

A study by Hirano et al. (26) analyzing 98 cases of EoG found that the most common endoscopic findings were erythema (72%), erosions (46%), and granularity (35%). Similarly, in the present study, the most frequently observed findings in EoG were erythema (42.9%), ulcer or erosions (23.8%), and granularity (19%), showing comparable results. However, in this study, pyloric stenosis was observed in 19% of cases, which is notably higher than the reported prevalence of 1.5% in Western studies. This finding suggests that pediatric patients may have a higher likelihood of pyloric muscle layer involvement compared to adults. However, as this hypothesis is based on a small-scale study, larger studies are warranted to validate these findings.

To date, no standardized therapeutic guidelines for non-EoE EGIDs have been firmly established. However, based on available guidelines, elimination diets have been suggested to improve symptoms and induce remission. Additionally, proton pump inhibitors (PPIs) may be considered for treating gastrointestinal ulceration in patients with EoG or EoD (4). Furthermore, biologic agents are currently under ongoing clinical trials to explore their potential as therapeutic options for EGIDs.

In the present study, PPIs were the most commonly used treatment in both EoE and non-EoE EGIDs, followed by tailored food elimination. Many patients in this study received combination therapy with PPIs and other treatment modalities. However, according to guidelines, the recommendation for combination therapy in non-EoE EGIDs remains neutral, except in cases where coexisting allergic diseases are present, in which case combination therapy is recommended (4). There are many cases reported in Korea where good responses to treatment with suspected food restrictions and/or ketotifen administration were observed (27).

Although systemic oral corticosteroids have been reported to improve both clinical symptoms and histologic inflammation in patients with non-EoE EGIDs, standardized guidelines regarding their indication, optimal dosage, and duration of therapy have yet to be established (4).

The present study has several limitations. Despite being a multicenter investigation, the relatively small sample size may limit the robustness and generalizability of our findings. In particular, all analyses were performed based on endoscopic procedure counts and not unique patient numbers. Because some individuals underwent both upper and lower endoscopies, the total number of procedures exceeds the number of individual children evaluated, and the prevalence and some clinical proportions may be slightly overestimated in comparison to true patient-based rates. We also acknowledge that we did not assess the potential impact of extreme fecal calprotectin values on our group comparisons, which remains a limitation of this study. Additionally, as this was a retrospective study, variations could exist among participating institutions regarding biopsy procedures and histopathological diagnostic criteria, even though patient registry was performed based on the newly established guidelines and nomenclature.

Nevertheless, this study is meaningful as it represents the first study in Korea to investigate the prevalence of EGIDs in pediatric patients, along with the clinical characteristics of EoE and non-EoE EGIDs, using the recently updated diagnostic criteria and nomenclature.

In summary, this study identifies a distinctive epidemiological profile of pediatric EGIDs in Korea, characterized by a greater prevalence of non-EoE EGIDs relative to EoE, in contrast to patterns reported in Western studies. Notably, non-EoE EGIDs with EI exhibited higher peripheral eosinophil counts and fecal calprotectin concentrations than EoE. These results suggest the potential value of these biomarkers in differentiating disease subtypes and in assessing their diagnostic and prognostic significance. Overall, our findings provide foundational data that may inform future research and the refinement of diagnostic strategies for EGIDs in Asian pediatric populations.

## Data Availability

The original contributions presented in the study are included in the article/Supplementary Material, further inquiries can be directed to the corresponding author.
